# Recent Development of Augmented Reality in Surgery: A Review

**DOI:** 10.1155/2017/4574172

**Published:** 2017-08-21

**Authors:** P. Vávra, J. Roman, P. Zonča, P. Ihnát, M. Němec, J. Kumar, N. Habib, A. El-Gendi

**Affiliations:** ^1^Department of Surgery, University Hospital Ostrava, 17. Listopadu 1790, 708 52 Ostrava, Czech Republic; ^2^Faculty of Medicine, University of Ostrava, Syllabova 19, 703 00 Ostrava, Czech Republic; ^3^Faculty of Electrical Engineering and Computer Science, Technical University of Ostrava, 17. Listopadu 15/2172, 708 33 Ostrava, Czech Republic; ^4^Department of Surgery & Cancer, Faculty of Medicine, Imperial College London, South Kensington Campus, London SW7 2AZ, UK; ^5^Department of Surgery, Faculty of Medicine, Alexandria University, Chamblion Street, El Azareeta, Alexandria Governorate, Egypt

## Abstract

**Introduction:**

The development augmented reality devices allow physicians to incorporate data visualization into diagnostic and treatment procedures to improve work efficiency, safety, and cost and to enhance surgical training. However, the awareness of possibilities of augmented reality is generally low. This review evaluates whether augmented reality can presently improve the results of surgical procedures.

**Methods:**

We performed a review of available literature dating from 2010 to November 2016 by searching *PubMed* and *Scopus* using the terms “augmented reality” and “surgery.” *Results*. The initial search yielded 808 studies. After removing duplicates and including only journal articles, a total of 417 studies were identified. By reading of abstracts, 91 relevant studies were chosen to be included. 11 references were gathered by cross-referencing. A total of 102 studies were included in this review.

**Conclusions:**

The present literature suggest an increasing interest of surgeons regarding employing augmented reality into surgery leading to improved safety and efficacy of surgical procedures. Many studies showed that the performance of newly devised augmented reality systems is comparable to traditional techniques. However, several problems need to be addressed before augmented reality is implemented into the routine practice.

## 1. Introduction

The first experiments with medical images date back to the year 1895, when W. C. Röntgen discovered the existence of X-ray. This marks the starting point of using medical images in the clinical practice. The development of ultrasound (USG), computed tomography (CT), magnetic resonance imaging (MRI), and other imaging techniques allows physicians to use two-dimensional (2D) medical images and three-dimensional (3D) reconstructions in diagnosis and treatment of various health problems. Further development of medical technology has given an opportunity to combine anatomical and functional (or physiological) imaging in advanced diagnostic procedures, that is, functional MRI (fMRI) or single photon emission computed tomography (SPECT/CT). These methods allowed physicians to better understand both the anatomical and the functional aspects of a target area.

The latest development in medical imaging technology focuses on the acquisition of real-time information and data visualization. Improved accessibility of real-time data is becoming increasingly important as their usage often makes the diagnosis and treatment faster and more reliable. This is especially true in surgery, where the real-time access to 2D or 3D reconstructed images during an ongoing surgery can prove to be crucial. This access is further enhanced by the introduction of augmented reality (AR)—a fusion of projected computer-generated (CG) images and real environment.

The ability to work in symbiosis with a computer broadens horizons of what is possible in surgery, as AR can alter the reality we experience in many ways. The wide range of possibilities it offers to surgeons challenges us to develop new techniques based on AR. In the future, AR may fully replace many items required to perform a successful surgery today, that is, navigation, displays, microscopes, and much more, all in a small wearable piece of equipment. However, the awareness of AR implementation and what it may offer is generally low, as at current state, it cannot fully replace most of long established surgical methods. The main aim of this work is to focus on the latest trends of the rapidly developing connection between augmented reality and surgery.

## 2. Methods

We performed a review of available literature dating from 2010 to November 2016 by searching *PubMed* and *Scopus* using the terms “augmented reality” and “surgery.”

The initial search yielded 808 studies. After removing duplicates and including only journal articles, a total of 417 studies were identified. By reading of abstracts, 91 relevant studies were chosen to be included. 11 references were gathered by cross-referencing. A total of 102 studies were included in this review.

## 3. Results and Discussion

### 3.1. Basic Principles of Augmented Reality

**Figure 1:**
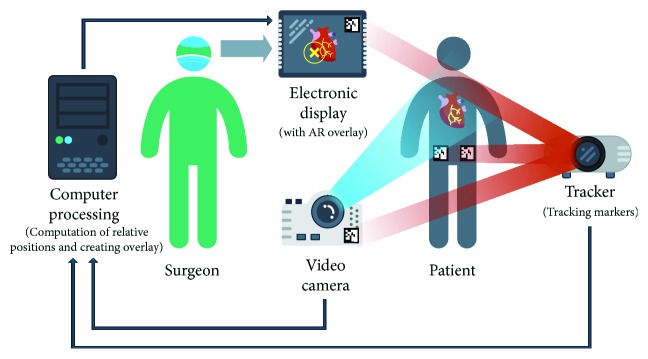
A scheme showing the basic principles of augmented reality.

An augmented reality system provides the surgeon with computer-processed imaging data in real-time via dedicated hardware and software. The projection of AR is made possible by using displays, projectors, cameras, trackers, or other specialized equipment. The main principle of a basic AR system is presented in Figure 1. The most basic method is to superimpose a CG image on a real-world imagery captured by a camera and displaying the combination of these on a computer, tablet PC, or a video projector [1–7]. In case it is impossible to mount a video projector in the operating room, a portable video projection device has been designed [3, 4]. The main advantage of AR is that the surgeon is not forced to look away from the surgical site as opposed to common visualization techniques.

Another possibility is to use a special head-mounted display (HMD, sometimes referred to as “smart glasses”) which resembles eyeglasses. They use special projectors, head tracking, and depth cameras to display CG images on the glass, effectively creating the illusion of augmented reality. Several AR systems with a HMD have already been developed with success [1, 8]. Using a HMD is beneficial as there is almost no obstruction in the surgeon's view compared to a traditional display; it is not necessary to move the display, and the need of a proper line-of-sight alignment between the display and the surgeon is not as accented [9].

At present, the applications of AR are limited by the essential requisite of preoperative 3D reconstructions of medical images. It is possible to create these reconstructions by using commercial or self-made software from the Digital Imaging and Communications in Medicine (DICOM) format [7, 10–12]. The quality of a reconstruction depends on the quality of input data and the accuracy of the reconstruction system. Such reconstructions can be used for virtual exploration of target areas, planning an effective surgical approach in advance, and for better orientation and navigation in the operative field.

The type and amount of displayed data rely on the requirements of the procedure and personal preferences of the surgical team. AR is especially useful in visualizing critical structures such as major vessels, nerves, or other vital tissues. By projecting these structures directly onto the patient, AR increases safety and reduces the time required to complete the procedure. Another useful feature of AR is the ability to control the opacity of displayed objects [13, 14]. Most HMDs allow the wearer to turn off all displayed images, becoming fully opaque, thus removing any possible distractions in an emergency. Furthermore, it is possible to utilize voice recognition to create voice commands, enabling hands-free control of the device. This is especially important in surgery as it allows surgeons to control the device without the need of assistance or break aseptic protocols. Another interesting option is to use gesture recognition, allowing the team to interact with the hardware even on sterile surfaces or in the air through body movements [6].

### 3.2. Monitoring the Operative Field

Most surgeries target deformable structures, which change significantly during a procedure (i.e., a removal of tissue during a resection). This problem needs to be addressed by constantly monitoring the operative field and making real-time changes to the displayed 3D model. It is possible to use perioperative ultrasound [15, 16], CT, or MRI to update the surgical site model. However, the amount of time required to capture and reconstruct medical images is significant. Using CT [17] or its modifications (i.e., C-arm cone-beam CT [18]) expose the patient to radiation and therefore can only be performed in a limited number of times. An open MRI is a feasible option for perioperative imaging; however, a single surgery requires 40 ± 9.4 minutes of scanning in average and the use of specialized MRI-compatible instruments [19]. Automatic medical reconstructions tend to include many different structures, which make the orientation difficult, especially in abdominal surgery. In some cases, this can be overcome by a method proposed by Sugimoto et al. [2]. In this technique, carbon dioxide is introduced into the gastrointestinal tract and pancreatico-biliary duct in conjunction with an intravenous contrast agent, which allows better display of these individual structures [2]. The quality of reconstructions may be improved in the future due to the development of imaging techniques, which allow making more detailed and refined images. Medical imaging may be replaced by depth-sensing cameras or video cameras in some cases; however, this method cannot detect structures beneath the surface that might be negated by developing a software to predict tissue behaviour by measuring forces applied to the target tissue. However, all currently proposed solutions need further improvement [7, 12, 17, 20, 21].

### 3.3. Augmented Reality for Education and Cooperation

Augmented reality proved to be an effective tool for training and skill assessment of surgery residents, other medical staff, or students [22–26]. Specialized training simulators can be created and used to improve surgeons' skills in various scenarios, as well as to objectively measure their technical skills. This will be especially beneficial for trainee residents and students in developing intuition and proper decision-making abilities, which can otherwise be only gained through long clinical practice. It allows simulation of very unlikely scenarios, which can be solved with the assistance of an experienced surgeon. Compared to virtual reality (VR) simulators, where the whole simulation takes part in a CG environment, the main advantage of AR simulators is the ability to combine real life objects with CG images, resulting in satisfactory tactile feedback as opposed to VR. AR has also been reported to increase the enjoyment of basic surgical training [27].

AR enables experienced surgeons to remotely assist residents by using Internet connection and therefore opens the way of excellent distant teaching. Shenai et al. [28] created virtual interactive presence and augmented reality (VIPAR) system. By continually monitoring and transmitting the image of a surgical site between two distant stations, it enables remote virtual collaboration between two surgeons [28]. This concept is sometimes referred to as “telepresence.” Davis et al. [29] used VIPAR system in an effort to allow communication between Vietnam and the USA. Thanks are due to the high resolution of the transmitted image, submillimetre achieved precision, and average latency of 237 milliseconds; the interaction between both surgeons was described as effective [29].

### 3.4. Methods of Image Alignment

The exact alignment of a real environment and CG images is an extremely important aspect to consider. The simplest method is to manually align both images [30]. Such a method is slow and may be imprecise, and therefore the registration process (the alignment of preoperative images with the currently treated patient) needs to be continuous to compensate for the changes in organ layout, that is, during breathing. The accurate alignment of both images is achieved by a set of trackers, which are used to determine the exact position of the camera and patient's body. These trackers usually track fiducial markers placed on the surface of specific structures, which remain still during the surgery (i.e., iliac crest, clavicles, etc.) and thus providing the system with points of reference.

There are several types of markers described in the literature. Many authors used a set of optical markers and a dedicated camera to detect them for navigation [15, 31–36]. The positions of the camera and the patient are determined by measuring distances between individual trackers and markers. Several studies reported using a set of infrared markers [4, 10, 13, 33, 37–40]. A newer approach suggested by Wild et al. [41] involved using fluorescent markers during laparoscopic procedures. The main advantage of such markers is the clear visibility even in difficult conditions and the ability to remain in the patient after the surgery. However, this requires an endoscopic system capable of detecting the emitted light [41]. Konishi et al. proposed an electromagnetic tracking system paired with an infrared sensor for laparoscopic surgery [42]. The main problem of magnetic tracking is the distortion caused by metal tools and equipment, which can however be minimized by using a special calibration technique [43]. The passive coordinate measurement arm (PCMA) method promises very high precision and the possibility to navigate without a direct line of sight by using a robotic arm for distance measurement [44]. Another method of registration is tracking of a laparoscopic camera and consequent automatic alignment of captured video with a 3D reconstruction [45]. Inoue et al. proved that using simple and affordable equipment, for instance a set of regular web cameras and freeware 3D reconstruction software, can be used to create a registration system thus depicting the purchase of such systems as possibility for all medical institutions [10]. The necessity of a direct line of sight may reduce the maximum possible organ deformation and rotation as well as the maximum range of tracked tools, depending on the exact configuration of the tracking system. It is possible to employ an increased number of tracker markers to minimize the chance of tracking failure due to a line of sight obstruction. Several commercially available tracking systems for medical AR are available, relying mostly on infrared or electromagnetic tracking [46].

With advancement in technology in the future, it may be possible to track organ position in real-time without the use of dedicated markers, using various methods for analysis of the operative field. Hostettler et al. used a real-time predictive simulation of abdominal organs [47], and Haouchine et al. designed a physics-based deformation model of registration [48]. These approaches are based on the usage of computing power to predict and visualize organ movement and deformation. It is also possible to use an RGB (red-green-blue) or range camera to perform registration without the use of markers [49, 50]. On the other hand, Hayashi et al. described natural points of reference as tracking points in the patient's body for progressive registration of cut vessels as markers [50]. Kowalczuk et al. created a system for real-time 3D modelling of the surgical site, with accuracy within 1.5 mm by using a high definition stereoscopic camera and a live reconstruction of the captured image [51].

Another possibility is to use a laser surface scanning technique, aligning images by scanning a high number of surface points without the use of fiducial markers. While Krishnan et al. found the accuracy of laser scanning to be sufficient [52], another study by Schicho et al. [53] suggests lower accuracy compared to using fiducial markers, with the overall deviation of 3.0 mm and 1.4 mm, respectively. Authors suggest that the accuracy may be improved by using an increased number of tracked surface points [53]. An additional marker-less registration method has been described by Lee et al., where authors combined a cone-beam CT image with a 3D RGB image with a sufficient accuracy of 2.58 mm; the absence of real-time image alignment is however still an issue [54]. All camera-based techniques are severely limited by the necessity of a direct line of sight. A novel method designed by Nosrati et al. [55] estimated organ movement by combining preoperative data, intraoperative colour, and visual cues with a vascular pulsation detection (also described by Amir-Khalili et al. [56]), resulting in a robust system not affected by common obstructions (light reflection, light smoke). This method increased the accuracy by 45% compared to traditional techniques. The proposed method, however, is not capable of real-time computation, as every registration requires approximately 10 seconds to be completed [55].

It needs to be noted that the amount of time required to prepare and calibrate an AR system needs to be considered for routine implementation. Pessaux et al. [12] reported a delay of a few seconds for each marker registration. The total time for AR-related registrations was 8 (6–10) minutes [7, 16]. The registration process of a marker-less system used by Sugimoto et al. [2] was completed within 5 minutes. The latency of displaying movement is important as lower latency generally means a better experience.

### 3.5. Precision

The accuracy and complexity of 3D reconstructed imaging are crucial in providing the correct data to the surgical team. An exact comparison of accuracy between specific studies is impossible due to variable conditions and different approaches for measuring accuracy. Optical systems feature precision within 5 mm in several studies [8, 10, 15, 18, 35, 37, 44, 57–59], which is considered sufficient for clinical application. The required precision differs greatly among various procedures and should be determined individually. The PCMA method of registering relative positions represents the best precision of all mentioned techniques, with an overall precision of <1 mm [44]. Yoshino et al. used a high-resolution MRI image for the reconstruction and an optical tracking system, with reported accuracy of 2.9 ± 1.9 mm while using an operative microscope during an experiment in a phantom model [31]. Two studies reported using a video projector during phantom experiments or an actual surgery, with results comparable to using an electronic display, featuring accuracy of 0.8–1.86 mm [1, 5]. Gavaghan et al. proposed a portable projection device with accuracy of 1.3 mm [3]. A few authors achieved a deviation of <2.7 mm while using a head-mounted display [8, 40, 60]. Although the difference in precision between a computer monitor and an HMD is not statistically significant [40]. It has been noted that the accuracy is not dependent on the surgeon's experience [8]. The maximum achievable precision is further diminished due to the difficulties in giving the projected image 3D appearance [1, 31] or giving a correct depth perception [8, 32, 34]. One of the possible solutions is to display objects with different opacity [61] or increasingly dark colour. This could be further minimized by taking advantage of motion parallax, which can be created by tracking surgeons head and modifying the projection accordingly [62].

### 3.6. Uses in Clinical Practice

Augmented reality can be used effectively for preoperative planning and completion of the actual surgery in timely fashion. The preoperative 3D reconstructed images can be modified and prepared for display in AR systems. Commonly, AR is used for tailoring individually preferred incisions and cutting planes [1, 36, 58], optimal placement of trocars, [63] or to generally improve safety by displaying positions of major organ components [7]. Another benefit of AR is the ability to aid surgeons in difficult terrain after a neoadjuvant chemotherapy or radiotherapy [64]. AR may be used to envisage and optimize the surgical volume of resection [16]. In many procedures, the AR-assisted surgery is comparable to other methods of assistance and the usage of such devices depends on surgeon's preference [58].

Augmented reality systems are the most useful during a surgery of organs with little movement and deformation (i.e., skull, brain, and pancreas) as the least amount of tracking and processing power is required whereas mobile organs, like the bowel, are significantly more complicated track and display. Considering these facts, the most frequently targeted areas in AR research are the head and brain [1, 8, 9, 11, 22, 31, 34, 36, 59, 61, 65–69], orthopaedic surgery [4, 37, 60, 70–77], hepato-biliary system [2, 3, 7, 13, 18, 32], and pancreas [13, 14, 35]. On top of that, AR may compensate the lack of tactile feedback usually experienced during laparoscopic surgery by presenting the surgeon with visual clues, thus improving hand-eye coordination and orientation, even in robotic surgery [78, 79]. Many studies proposed using augmented reality in laparoscopic procedures with success [7, 33, 38, 51, 64, 69].

Neurosurgical procedures have employed AR systems successfully, because of the inherent limitation of head and brain movement. It has been reported that AR had a major impact in 16.7% of neurovascular surgeries [34], allowing a higher rate of precise localization of lesions and shorter operative time compared to a traditional 2D approach [66]. Neurosurgeons benefit mainly from precise localization of individual gyri, blood vessels, important neuronal tracts, and the possibility to plan the operation corridor, for instance, in a removal of superficial tumours [10, 66], epilepsy surgery [65], or in neurovascular surgery [80].

AR has also proved to be useful during orthopaedic surgeries and reconstructions, especially because it allows to view reconstructions directly on top of the patient's body, which reduces the number of distractions caused by looking at an external display [37]. The list of procedures successfully utilizing AR ranges from minimally invasive percutaneous surgeries to trauma reconstructions [74], bone resections, osteotomies [73, 77, 81], arthroscopic surgery, Kirschner wire placement [82], joint replacement, or tumour removal [75, 76]. Percutaneous interventions only require a surface indicator of the insertion point, which can be displayed by AR [4]. A fluoroscopic dual-laser-based system was used by Liang et al. [37] for insertion guidance with satisfactory accuracy. However, the inability to perceive the depth of insertion forces the use of additional techniques [37]. In spite of that, the use of AR limits the radiation exposure during fluoroscopy and the amount of time required to perform the task [71, 72] while minimizing the risk of unnecessary haemorrhage. Rodas and Padoy used AR to create a user friendly visualization of scattered radiation during a minimally invasive surgery [70], enabling to measure and visualize the amount of radiation received. AR can change the workflow of creating orthopaedic implants, replacing 3D printing of a patient-specific model [60]. It is also possible to employ AR in orthopaedic robot-assisted surgeries [81].

It is more difficult to use AR in abdominal surgery, as the amount of organ movement is significant; nevertheless, it is currently used during liver and pancreatic surgeries as it allows better projection of large vessels [39] or tumour sites owing to comparative static nature of these organs. By comparing the reconstructed data with intraoperative ultrasound, AR can be used for intraoperative guidance during liver resections [83]. It can also assist the surgeon with the establishment of laparoscopic ports [13] or phrenotomy sites [84]. Similarly, the kidneys appear to be suitable for the usage of AR, as demonstrated by Muller et al. during a nephrolithotomy, where the AR was used to establish the percutaneous renal access [58]. An AR system has been proposed to accurately detect a sentinel node using a preoperative SPECT/CT scan of the surrounding lymph nodes. This allows to precisely navigate to the sentinel node and perform a resection even in difficult terrain [85]. Using a freehand SPECT to scan radiation distribution, reconstruction of the data and its implementation into AR in real time can extend the concept [27]. AR has also been successfully employed during splenectomies in children [33] and urological procedures [86]. With the increasing precision of AR systems, they can be safely used even in endocrine surgeries [87–89], otorhinolaryngologic surgery [90], eye surgery [91], vascular surgery [92, 93], or dental implantology too [60]; however, their exact usefulness in such surgeries is not exactly quantified owing to the complicated structures [61]. An AR system for transcatheter aortic valve implantation was designed and successfully used by Currie et al., showing comparable accuracy to traditional fluoroscopic guidance technique, hence avoiding impending complications of contrast agent administration [93].

The use of AR in robotic surgery is expanding rapidly, due to its ability to easily incorporate AR directly into the operators' console, which allows the surgeon to navigate more quickly and better identify important structures [7, 94]. The CG projection can be displayed separately or as an overlay of real-time video as needed by the surgeon. A few authors successfully applied AR during a robotic surgery with satisfactory results, showing a possible role of AR as a future trend in robotic surgery [12, 79, 95].

### 3.7. Advanced Image in Fusion Using Augmented Reality

Augmented reality cannot only display CG images but can also display images which are not normally visible. A handful of studies successfully combined optical images with near-infrared fluorescence images [96–99]. This technique can be used to increase precision by providing additional data for a surgeon to consider. Using this technique, surgeons could be able to detect blood vessels under the organ surface or detect other tissue abnormalities. Koreeda et al. [100] proposed another interesting concept. Here, AR has been used to visualize areas obscured by surgical instruments in laparoscopic procedures, making the tools appear invisible. This system also helped in effectively reducing the needle exit point errors during suturing, while achieving average accuracy of 1.4 mm and acceptable latency of 62.4 milliseconds [100].

### 3.8. Problems of Augmented Reality

Augmented reality introduces many new possibilities and adds new dimensions to surgical science. Surgeons can use additional data for decision making and improving safety and efficacy. Advances in technology allow AR devices to display information with increasing accuracy and lower latency. Despite of constant improvement, there are several difficulties which need to be addressed. Currently, all reconstructed images need to be prepared in advance using complex algorithms requiring powerful computers. However, thanks to an expected advancement in technology, a real-time acquisition of high-resolution medical scans and 3D reconstructions may be possible. Such reconstructions of the operative field would significantly improve overall accuracy. Even though AR can speed up a surgical procedure, the necessity to prepare the whole system and make required registrations and measurements generally increases the time necessary to complete the surgery, with the amount of time depending on the type of the procedure and the complexity of the AR system. The introduction of fully automatic systems would eliminate this problem and reduce the total time required for completion.

The time required for completing a procedure has been reduced while using any form of AR; however, there are certain limitations. One of them is inattentional blindness (an event where the surgeon does not see an unexpected object which suddenly appears in his field of view), which is a concerning issue that needs to be addressed while using 3D overlays [101]. The amount of information a surgeon has presented through AR during a surgery is increasing and may be distracting [60]. Therefore, it is necessary to display only important data or provide a method to switch between different sets of information on demand. A method for the reduction of cognitive demands was proposed by Hansen et al. [102]. By optimizing the spatial relationship of individual structures, reducing the occlusion caused by AR to minimum, and maximizing contrast, surgeons were able to reduce visual clutter in some cases [102]. Reaching an adequate image contrast during a projection directly onto organs is also necessary [13]. Difficulties in creating a correct 3D and depth perception also persist. The latency of the whole system is also of concern because excessive latency may lower precision and reduce comfort of the surgeon. Kang et al. measured the latency of their optical system for laparoscopic procedures to be 144 ± 19 milliseconds [15].

Currently used head-mounted displays usually weigh several hundred grams and produce plenty of heat; therefore, long-term wear comfort is an issue. These need to be addressed in future to better fit the ergonomics and allow continuous usage for a long period. It is known that virtual and augmented reality projections in HMDs produce simulator sickness, which is presented by nausea, headache, and vertigo or vomiting in the worst scenario. The exact causes behind the simulator sickness are unknown; however, a discrepancy between visual, proprioceptive, and vestibular inputs is probably the case.

## 4. Conclusions

Studies suggest that AR systems are becoming comparable to traditional navigation techniques, with precision and safety sufficient for routine clinical practice. Most problems faced presently will be solved by further medical and technological research. Augmented reality appears to be a powerful tool possibly capable of revolutionising the field of surgery through a rational use. In the future, AR will likely serve as an advanced human-computer interface, working in symbiosis with surgeons, allowing them to achieve even better results. Nevertheless, further advancement is much needed to achieve maximum potential and cost-effectiveness of augmented reality.
